# Incremental Value of Digital PET/MRI over PET/CT in the Assessment of Neoplastic Liver Lesions

**DOI:** 10.1055/s-0045-1814145

**Published:** 2025-12-18

**Authors:** Pawan Gulabrao Shinkar, Mohana Vamsy, Dileep Kumar, Palak Wadhwa

**Affiliations:** 1 Department of Radiology and Nuclear Medicine, Omega Hospitals, Hyderabad, Telangana, India; 2 Department of Surgical Oncology, Omega Hospitals, Hyderabad, Telangana, India; 3 United Imaging Healthcare, Shanghai, People's Republic of China

**Keywords:** PET/MRI, liver lesions, molecular imaging, multimodality scanning, neoplastic hepatic lesions, PET/CT

## Abstract

**Objectives:**

The objective of this study was to assess the performance of positron emission tomography/magnetic resonance imaging (PET/MRI) compared with PET/computed tomography (PET/CT) in the clinical management of patients with neoplastic hepatic lesions.

**Materials and Methods:**

This is a retrospective study and includes a sample size of 15 patients, referred for diagnostic evaluation and staging of neoplastic hepatic lesions. The patients included in this study underwent a simultaneous PET/CT scan on uMI-Vista and a complementary liver PET/MRI scan on uPMR 790. PET/CT and PET/MRI were compared based on the number of detected lesions, the smallest detected lesion diameter, and tumor-to-liver ratio (TLR). The histopathological analysis was considered the standard of reference.

**Results:**

PET/MRI reported extra information in 87% (13/15) of patients, and additional lesions were identified in 73% (11/15) of patients. Furthermore, PET/MRI could identify subcentimeter liver lesions and added great value in the evaluation of lesion viability. Overall, 40 additional lesions were detected with PET/MRI in contrast with PET/CT within the given patient cohort. The smallest revealed lesion measured 2 mm in the long-axis diameter, and the average long-axis diameter of small lesions detected by PET/MRI across 15 patients was 3.4 mm with a standard deviation of 1.3 mm. These findings significantly affected the final outcomes in 12 out of 15 patients, leading to modifications in the response assessment category in 5 patients and defined the malignant hepatic lesions on staging/restaging scans (10/15).

**Discussion:**

PET/MRI has been found to outperform PET/CT in terms of conspicuity of liver lesions, with better sensitivity and specificity. Overall, coregistered PET and MR images have been shown to outperform PET/CT in the imaging of liver lesions, with better delineation of small lesions as well as reliable localization of lesions to the corresponding liver segment.

**Conclusion:**

In addition to a significant decrease in radiation exposure, the PET/MRI combination resulted in higher detection rates and more precise characterization of small malignant liver lesions and tends to be more powerful than PET/CT, which has a direct impact on the patient's diagnosis, staging, and further therapeutic strategies.

## Introduction

Neoplastic liver lesions present significant diagnostic challenges due to their diverse etiology, variable morphological features, and overlapping imaging characteristics. 1 Accurate characterization and staging of these lesions are critical for guiding optimal treatment strategies and improving patient outcomes. 2 In recent years, hybrid imaging modalities, such as positron emission tomography/magnetic resonance imaging (PET/MRI) and PET/computed tomography (PET/CT), have emerged as invaluable tools for the comprehensive evaluation of neoplastic liver lesions. 3

PET/CT has become a cornerstone in oncological imaging, providing sequential metabolic and anatomical information. 4 The fusion of functional PET data with high-resolution CT images enables precise localization, characterization, and staging of neoplastic liver lesions, thereby facilitating treatment planning and monitoring of therapeutic response. 5 However, soft tissue contrast of CT is not optimal for diagnosis and differentiation of soft tissue lesions. The PET images are also affected by the number of factors including age, blood sugar, body mass index incubation time, and hepatic steatosis affects the liver capacity to absorb fluoro-D-glucose (FDG).

Furthermore, since PET is not a standalone modality and requires anatomical localization and limits the detectability of small size and low contrast lesions, PET on its own is not suitable for the detection of neoplastic hepatic lesions. Clinically significant lesions may be missed by PET/CT due to the aforementioned limitations.

On the other hand, PET/MRI combines the metabolic insights of PET with the superior soft tissue contrast, enhanced spatial resolution, and multiparametric imaging capabilities of MRI. 6 This hybrid modality offers several potential advantages for the assessment of neoplastic liver lesions, including enhanced soft tissue characterization, improved intrinsic contrast resolution with reduced injected contrast agent, reduced radiation exposure, and truly simultaneous acquisition providing functional, anatomical, and molecular information. 7 In patients with renal impairment, noncontrast PET/MRI is superior to noncontrast PET/CT.

Despite the growing interest in PET/MRI for liver imaging, its comparative efficacy and diagnostic accuracy relative to PET/CT remain areas of ongoing investigation. 8 While PET/MRI holds promise for improving lesion detection and characterization, challenges such as motion artifacts and limited availability hinder its widespread clinical adoption. 9

This study aims to provide a comparative evaluation of hybrid PET/MRI and PET/CT for neoplastic liver lesions. 10 By synthesizing current evidence, discussing technical considerations, and evaluating diagnostic performance, this study seeks to elucidate the strengths, limitations, and clinical implications of each modality in liver lesion evaluation. 11 Furthermore, through a critical examination of emerging research and future directions, this research aims to contribute to optimized diagnostic algorithms and personalized treatment strategies for patients with neoplastic liver lesions. 12

## Materials and Methods

### Place of Study

The study was conducted at Omega Hospital, Hyderabad, India.

### Study Type

This study was a retrospective single-center observational cross-sectional study.

### Time Period

The data of this study were collected from January 2023 to May 2024.

### Study Population

This study included patients diagnosed with either primary or secondary liver neoplasms. Among primary liver tumors, hepatocellular carcinoma (HCC) is the most common, characterized by tumor cells resembling hepatocytes. HCC is strongly associated with chronic viral hepatitis and cirrhosis of various origins. Other primary liver tumors in this study include benign neoplasms associated with chronic liver parenchymal disease and cholangiocarcinoma. Cholangiocarcinoma, a primary adenocarcinoma originating from the bile ducts, resembles adenocarcinomas in other tissues, requiring exclusion of extrahepatic origins and differentiation from benign biliary lesions for accurate diagnosis.

Secondary liver neoplasms encompass metastatic liver lesions, which arise from cancers spreading to the liver from other parts of the body. The malignancies included in this study with liver metastases are colorectal cancer, breast cancer, neuroendocrine tumor, leiomyosarcoma of the uterus, and carcinoma vulva.

The inclusion criteria were as follows:

1. Patients of age older than 18 years with an ability to understand and hear instructions, and remain still for ∼20 minutes (the duration of PET/MRI scan).
2. Patients with an ability to undergo a PET/MRI scan within 30 minutes after the completion of a PET/CT scan.
3. Patients with a body weight of less than 200 kg.

The exclusion criteria were as follows:

1. Pregnant women.
2. Patients with metallic, conductive, electrically, or magnetically active implants not labeled as MRI-safe.
3. Patients with implants labeled as MRI-unsafe.

**Fig. 1:**
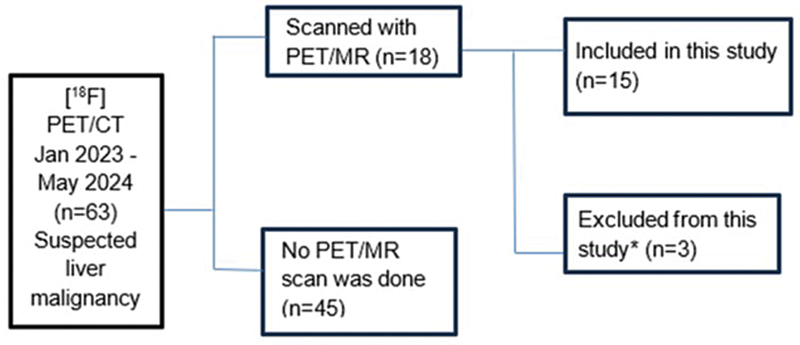
This figure shows the patient recruitment process: 63 patients who were suspected for liver malignancy between January 2023 and May 2024 underwent PET/CT scan. Forty-five patients who underwent PET/CT for a suspected liver malignancy did not agree to undergo the complementary abdomen PET/MRI scan. Three patients who underwent PET/MRI scan were excluded from the study due to movement, metallic artifacts, lack of cooperation, and gross ascites. CT, computed tomography; MRI, magnetic resonance imaging; PET, positron emission tomography.

All patients with suspected or histology-proven neoplastic liver lesions who were referred to undergo a PET/CT scan either for diagnosis, staging, or posttreatment staging and follow-up as treatment response evaluation, and those who passed the inclusion criteria as stated earlier, were offered to undergo a complementary liver PET/MRI scan by the institution. Out of 63 patients who underwent a PET/CT scan, only 18 patients agreed to undergo a complementary PET/MRI scan. The remaining 45 patients did not undergo a complementary PET/MRI scan due to the following reasons: claustrophobia, additional scanning time, and inconvenience. Further, out of 18 patients who underwent PET/MRI, only 15 were included in this study and 3 patients who underwent both PET/CT and a complementary liver PET/MRI scan were not included in this study due to movement, metallic artifacts, lack of cooperation, and gross ascites. The recruitment process is summarized in Fig. 1 .

Finally, the study population of this study included all patients affected with neoplastic hepatic lesions as primary or secondary malignancy, as described earlier and those who were successfully scanned with PET/CT and a complementary liver PET/MRI scan.

## Study Method

In this retrospective study, we included 15 patients with neoplastic hepatic lesions. All of these patients were referred for diagnostic evaluation and staging. The patients underwent simultaneous 2-deoxy-2-[18F]fluoro-D-glucose ([ ^18^ F]FDG]), [ ^68^ Ga] DOTANOC scan, and a complementary liver [ ^18^ F] FDG or [ ^68^ Ga] DOTANOC PET/MRI scan.

### PET/CT Scanning

Before the PET/CT scanning, patients were instructed to fast for at least 4 hours prior to the [18F] FDG injection. In all cases, the serum glucose concentration met the institutional requirement (≤140 mg/dL). Scanning was started 45 to 60 minutes after administration of 148 to 185 MBq of radiotracer.

PET/CT imaging was performed using a uMI Vista sequential digital PET/CT scanner (United Imaging Healthcare, Shanghai, China) over an axial field of view (FOV) from the apex of the skull to the mid-thigh. A whole-body contrast-enhanced CT scanning over 20s was performed first (120 kV, 200 mA, 0.8 seconds per CT rotation, pitch of 1.375:1, and table speed of 27.55 mm/second), 0.55 mm slice thickness with reconstruction interval of 1.0 mm with standard reconstruction kernel with additional breath hold CT for evaluation of the lungs. Digital PET scanning was performed immediately after acquisition of the CT images, without changing the patient position. Due to the digital nature of the scanner, the scanning time and injected dose were low, and the imaging was performed with five bed positions, with an acquisition time of 1 minute for each bed position. A 15% overlap in a 15.7 cm axial FOV and a 192 × 192 matrix size were used for image reconstruction. The emission data were corrected along with scattering, random, and decay correction.

### PET/MRI Scanning

After digital PET/CT imaging, all patients were imaged with simultaneous digital PET/MRI on the uPMR 790 PET/MRI system (United Imaging Healthcare), composed of a 3.0-Tesla MR scanner and a fully integrated time-of-flight digital PET scanner.

After performing a partial-body MR localizer scan, PET liver imaging was initiated, and data were collected for 20 minutes for one bed position. A PET scan was conducted with a FOV of 600 mm and with a reconstruction matrix size of 192 × 192 mm ^2^ . Along with PET, a newly customized liver MR protocol was devised for the efficient scanning of the patients. This protocol included MRAC sequence (a 3D T1-weighted spoiled gradient-echo sequence with Dixon-based water-fat separation imaging (WFI), T2 SSFSE/HASTE sequence, T2 FSE FAT SAT, and diffusion-weighted imaging (DWI) EPI. The plane in which sequence was acquired, repetition time (TR), echo time (TE), flip angle (FA), fat saturation (FS), respiratory trigger (RT), breath-hold (BH), free breathing (FB), phase FOV and readout FOV (FOV P + RO), slice thickness (Sl. Th.), slice gap, and acquisition time (Acq T) are all detailed in Table 1 and Supplementary Material S1. Compressed sensing-based technology was used to speed up the acquisition. Tissue segmentation and μ-map calculation were performed automatically by the vendor-provided algorithm.

**Table 1:** Tailored protocol for PET/MRI used for delayed liver imaging

Sequence	Plane	TR (ms)	TE (ms)	FA	FS	RT/BH/FB	Matrix	FOV P + RO (mm)	Sl. Th. (mm)	Gap (mm)	Acq T
T1 Dixon WFI	Axial	3.58	1.5	10°	None	RT+	288 × 75	300 × 400	5	1.5	3 min
T2 SS FSE/HASTE	Axial	1,100	92	120°	None	BH	304 × 100	300 × 380	4	0.5	55 s
T2 FSE	Axial	2,069	99	90°	FS	RT+	272 × 75	300 × 380	4	0.5	4 min
DWI EPI (B-value 0, 50, 600, 800)	Axial	8,651	64	90°	None	FB	112 × 100	300 × 380	3	0.5	4 min
T1 Dixon Quick 3D	Axial	3.58	1.57	10°	FS	BH	304 × 85	300 × 400	3.5	1.5	16 s
**Intravenous administration of 0.5 mmol/mL/kg body weight of gadoteric acid (a macrocyclic gadolinium-based contrast agent)**											
**Postcontrast sequence**							**Acq T**					
Noncontrast							16 s					
Early arterial phase							16 s					
Late arterial phase							16 s					
Portal venous phase							16 s					
Venous axial							16 s					
Venous coronal							16 s					
Delayed phase (3–5 min)							16 s					

Abbreviations: Acq T, acquisition time; BH, breath hold; FA, flip angle; FB, free breathing; FOV P + RO, phase field of view and readout field of view; FS, at saturation; MRI, magnetic resonance imaging; PET, positron emission tomography; RT, respiratory trigger; SI. Th., slice thickness; TE, echo time; TR, repetition time.

Note: MR sequences along with the duration demonstrate a short-duration PET/MRI scan, which is specifically designed for patient comfort.

For the dynamic contrast study, a T1 DIXON quick 3D breath-hold sequence was conducted before and after intravenous administration of 0.5 mmol/mL/kg body weight of gadoteric acid (a macrocyclic gadolinium-based contrast agent).

A multiphasic protocol conducted after the Gd contrast agent was injected intravenously and a detailed postcontrast protocol is described in Table 1 and Supplementary Material S1.

The entire MR protocol was conducted in conjunction with a quantitative PET enabled efficient evaluation of hepatic lesions with a PET/MRI scanner in a short time duration of 20 minutes. The detailed MR parameters are shown in Table 1 . The body array coil was placed around the individual and covered the upper abdomen. Respiratory gating and breath-hold techniques were used in MR acquisition whenever possible.

### PET/CT Image Reconstruction

Reconstruction was performed using a 3D ordered-subset expectation maximization (OSEM) algorithm with HYPER Deep Progressive Reconstruction (an AI-based PET reconstruction trained on high-count and total-body PET data for quantitative accuracy) with 2 iterations and 20 subsets algorithm. Attenuation correction was performed with CT data for the PET/CT protocol.

### PET/MR Image Reconstruction

Reconstruction was conducted with a 3D OSEM algorithm. A four-compartment-model attenuation map (μ-map) was automatically generated based on a water-fat-imaging sequence with breath gating and used for attenuation correction for the PET/MR protocol.

### Image Analysis

Two accredited and highly experienced readers with over 10 years of experience in hybrid and MRI analyzed the digital PET-CT and PET-MRI datasets.

Malignant lesions were classified on PET/CT scans, displaying areas of increased radiotracer uptake, with corresponding changes in density observed on the CT scan.

In PET/MRI scans, lesion characterization was performed based on all available T1 and T2 weighted as well as DWI sequences. Lesions were classified as malignant when at least two of the following criteria in MRI and PET were found: (1) potential restriction on DWI sequence, (2) hyperintense lesion in T2W sequence with ill-defined borders/target appearance, (3) gadolinium-based contrast enhancement pattern not in keeping with hemangioma/cyst, and (4) positive result on PET scan.

For each detected lesion, the maximum standardized uptake value (SUV _max_ ) was calculated in the corresponding PET modality. The number of lesions detected with PET/MRI was compared with PET/CT. Further, the lesion diameter was measured using the long-axis diameter on T2WI MR images, and further the smallest detected lesion diameter was recorded to demonstrate the potential of PET/MRI over PET/CT in detecting subcentimeter lesions. The tumor-to-liver ratio (TLR) was determined based on a measurement of the liver background SUV _max_ . The lesions detected on PET/CT and PET/MRI were paired according to the relative liver location, whenever possible.

## Results or Findings

### PET/MRI Detected More Liver Lesions

**Fig. 2:**
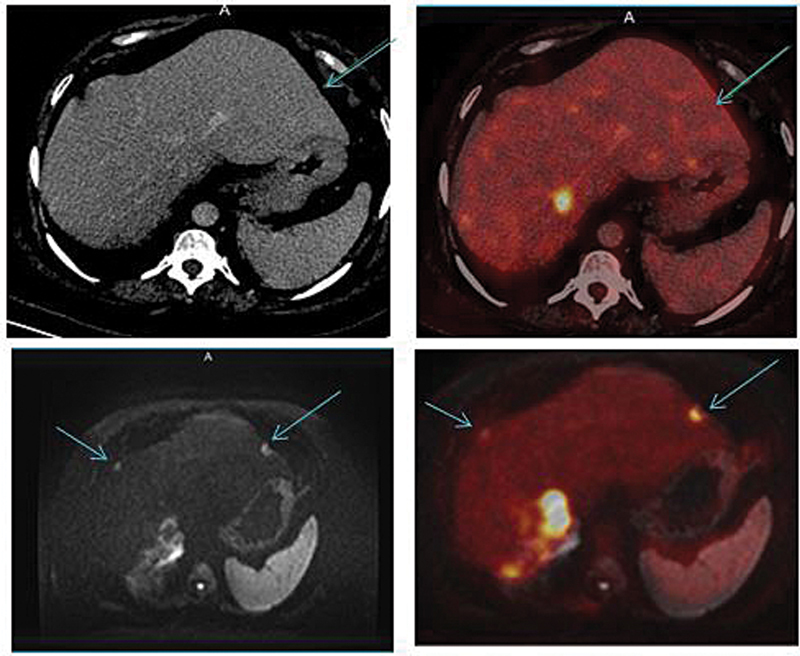
A 67-year-old man with cholangiocarcinoma, showing rising carbohydrate antigen 19-9 (Ca 19-9) levels (tumor marker for cholangiocarcinoma) with [ ^18^ F] FDG PET/CT scan (top row) and [ ^18^ F] FDG PET/MRI scan (bottom row). PET/CT revealed static disease, so for the better evaluation, PET/MRI of the liver was performed, which showed additional lesion in the left lobe of the liver (as can be seen with blue arrows in bottom row which was not well visualized in PET/CT). CT, computed tomography; FDG, fluoro-D-glucose; MRI, magnetic resonance imaging; PET, positron emission tomography.

**Fig. 3:**
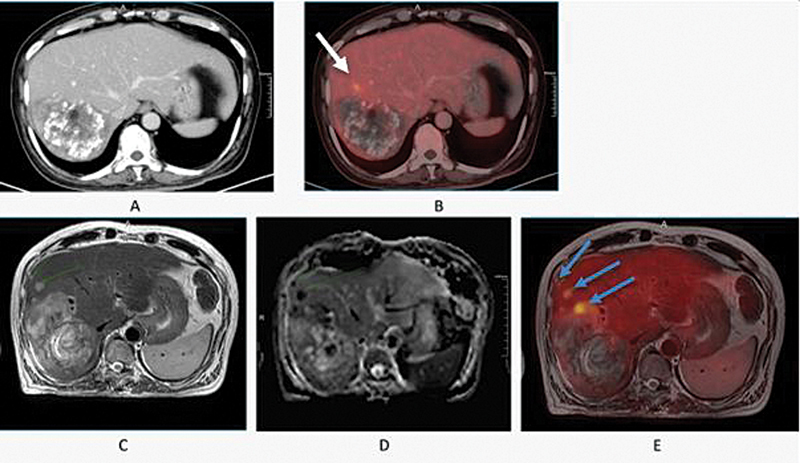
A 66-year-old man with hepatocellular carcinoma, showing a large hepatic mass with necrosis, radio-dense foci (post-TARE status), heterogeneous peripheral enhancement, and adjacent mild FDG uptake anteriorly as can be seen in PET/CT images ( **A** ) and ( **B** ). Axial T2W images ( **C** ) of [18F] FDG PET/MRI scans revealed a large hepatic mass showing heterogeneous FDG uptake in fused images with two small FDG avid nodular T2 mildly hyperintense lesions anterolateral to hepatic mass. ( **D** ) ADC sequence showing two small lesions showing hypointense signals. ( **E** ) PET/MRI showing two small adjacent lesions in segment VIII which was not visible in PET/CT image. ADC, apparent diffusion coefficient; CT, computed tomography; FDG, fluoro-D-glucose; MRI, magnetic resonance imaging; PET, positron emission tomography.

Coregistered PET and unenhanced MR images have been shown to outperform contrast-enhanced PET/CT in the imaging of PET-positive liver lesions. Figs. 2 and 3 demonstrate that PET/MRI helps with the detection of additional liver lesions over a PET/CT scanner, owing to the better soft tissue contrast, spatial resolution, and functional imaging capabilities of MRI such as DWI and apparent diffusion coefficient (ADC). Fig. 2 demonstrates a case of cholangiocarcinoma where additional liver lesions in the segment VIII and left lobe of liver were detected in PET/MRI and those lesions were not well appreciated in the PET/CT scan. Fig. 3 shows the capability of PET/MRI in the detection of additional subcentimeter lesions due to better spatial resolution and multiparametric sequence of MRI which were not visible in PET/CT.

### PET/MRI Shows Better Lesion Delineation Compared with PET/CT

Not only does PET/MRI help reveal additional PET-positive liver lesions but it has also supported better lesion delineation as compared with PET/CT, which further improves patient treatment management.

**Fig. 4:**
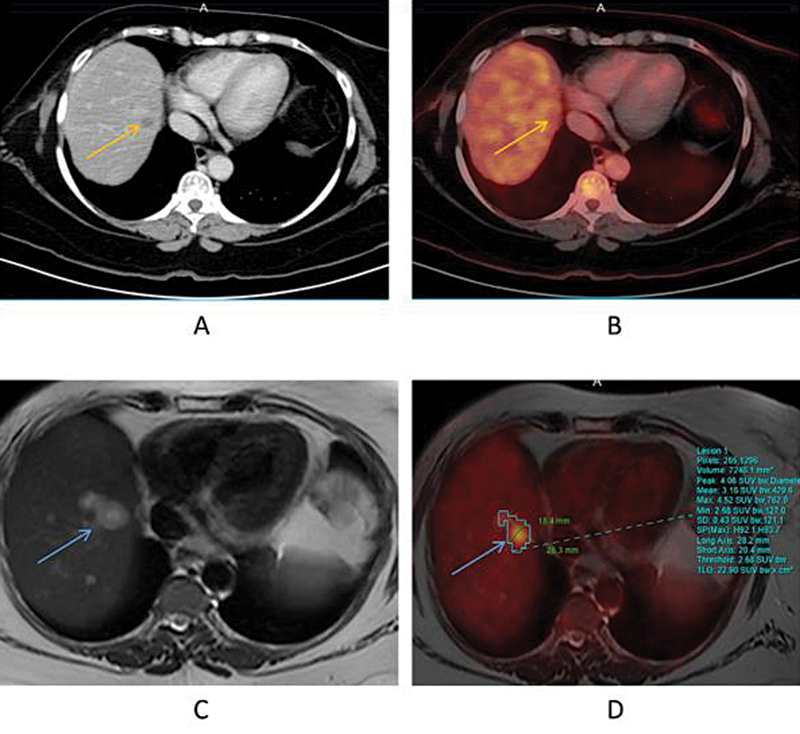
Case of breast cancer with liver metastases: Axial CT and simultaneous PET/CT images are shown in ( **A** ) and ( **B** ), respectively, and axial T2W MR and simultaneous PET/MRI are shown in ( **C** ) and ( **D** ), respectively, with improved lesion delineation seen in PET/MR images, highlighted by blue arrows. PET/MR images show bilobed T2 hyperintense lesion (blue arrow) in segment VIII of liver with FDG uptake which was minimal and vague on PET/CT images ( **A, B** ), highlighted with an orange arrow. CT, computed tomography; FDG, fluoro-D-glucose; MRI, magnetic resonance imaging; PET, positron emission tomography; T2W, T2-weighted.

A case of breast carcinoma with liver metastases demonstrated the advantage of PET/MRI over PET/CT owing to better lesion delineation with MR images. This can be seen in Fig. 4 , where T2W MR images have demonstrated improved lesion anatomical delineation as compared with the CT images.

### PET/MRI Reveals Additional Information over PET/CT

**Fig. 5:**
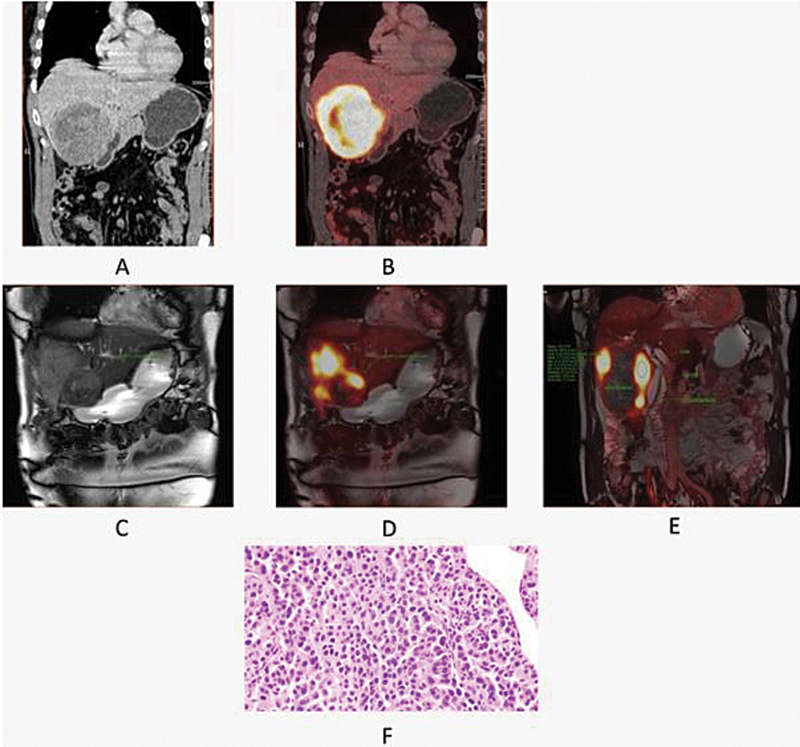
Case of hepatocellular carcinoma abutting adjacent structures: This figure shows the advantage of PET/MRI in revealing additional information about the hepatic mass as compared with PET/CT. ( **A** ) and ( **B** ) show coronal CT and simultaneous PET/CT images, respectively; ( **C** ), ( **D** ), and ( **E** ) show coronal T2W HASTE MR and simultaneous PET/MR images, respectively; ( **F** ) shows the histopathology image from true-cut biopsy of FDG avid portion of hepatic lesion. Coronal PET/CT images, ( **A** ) and ( **B** ), reveal a large hepatic mass predominantly in the right lobe and segment IV-B with partial exophytic component. Coronal PET/MR images ( **C–E** ) showing relation of large hepatic mass with the adjacent antrum of stomach, CHD, CBD, gallbladder and duodenum. ( **F** ) Histopathology and immunohistochemistry reports revealed neoplastic cells arranged in nests and sheets with cells showing pleomorphic hyperchromatic nuclei with mitosis, necrosis, and high Ki-67 index (75–80%). These features are consistent with hepatocellular carcinoma. CBD, common bile duct; CHD, common hepatic duct; CT, computed tomography; FDG, fluoro-D-glucose; MRI, magnetic resonance imaging; PET, positron emission tomography.

A case of HCC has shown further the benefit of PET/MRI where PET/MRI has revealed additional information in the form of relation of hepatic mass with the adjacent organs as compared with PET/CT. As can be seen in Fig. 5A, B , coronal PET/CT reveals a large hepatic mass predominantly in the right lobe and segment IV-B with partial exophytic component. Further, coronal PET/MR images as in Fig. 5C–E showed relation of large hepatic mass with the adjacent antrum of stomach, common hepatic duct (CHD), common bile duct (CBD), gallbladder, and duodenum. This information was not evident on PET/CT; the additional findings from PET/MRI supported more accurate patient management.

**Fig. 6:**
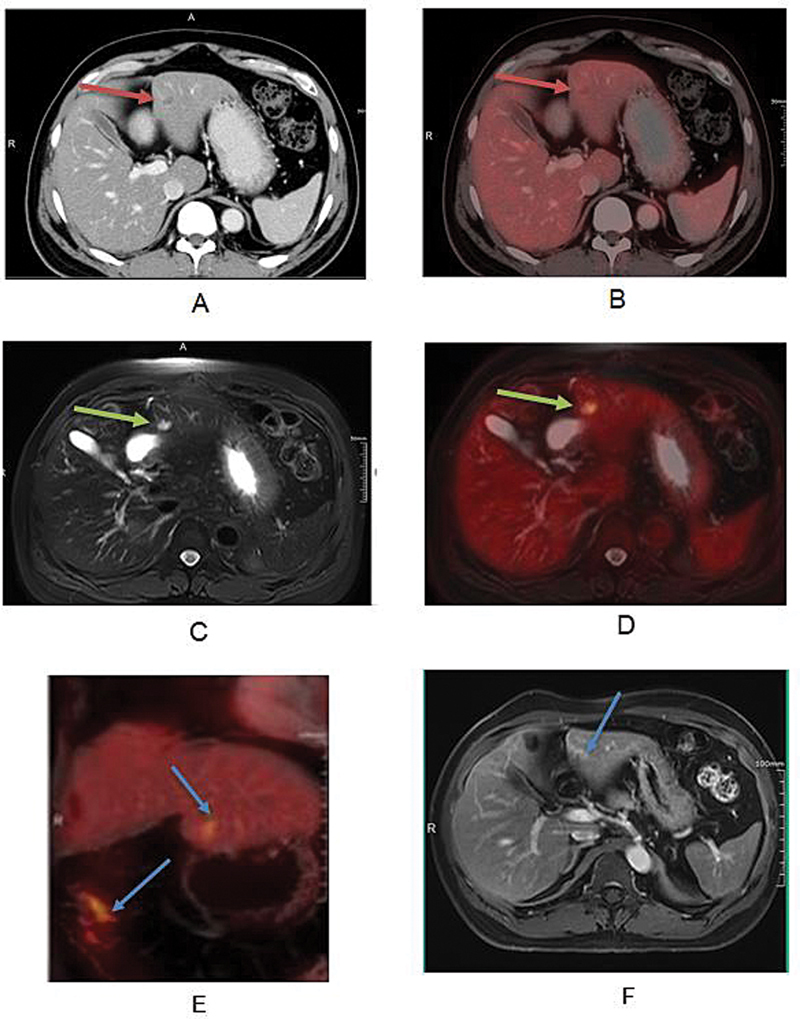
A 48-year-old man with colonic mass and liver lesions: This figure shows the advantage of PET/MRI in revealing additional information about the hepatic mass compared with PET/CT. ( **A** ) and ( **B** ) show axial CT and simultaneous PET/CT images, respectively; ( **C** ) and ( **D** ) show axial T2 FSE FS MR and simultaneous PET/MR images, respectively; ( **E** ) and ( **F** ) show fused contrast-enhanced PET/MRI and venous-phase MR images. PET/MRI shows lesion in segment IV of the liver with mild FDG uptake due to longer PET acquisition time and better spatial resolution as can be seen in ( **C** ) and ( **D** ). Further, DCE PET/MRI showed FDG avid peripherally enhancing hepatic lesion and FDG avid colonic lesion which was not visible in PET/CT as can be seen in ( **E** ). CT, computed tomography; DCE, dynamic contrast-enhanced; FDG, fluoro-D-glucose; MRI, magnetic resonance imaging; PET, positron emission tomography.

Fig. 6 also highlights the advantage of PET/MRI in providing additional information about the hepatic mass compared with PET/CT. PET/MRI reveals a lesion in segment IV of the liver with mild FDG uptake, attributed to longer PET acquisition time and better spatial resolution, as seen in Fig. 6C, D . Additionally, dynamic contrast-enhanced PET/MRI demonstrates an FDG-avid peripherally enhancing hepatic lesion and an FDG-avid colonic lesion, which were not visible on PET/CT ( Fig. 6E ).

### Quantitative Comparison between PET/MRI and PET/CT

In this study, a newly customized liver MR protocol was implemented in conjunction with quantitative PET to evaluate hepatic lesions using PET/MRI within a concise time frame of 20 minutes. The utilization of PET/MRI yielded additional information in 87% of cases and identified extra lesions in 73% of patients. PET/MRI exhibited improved lesion delineation compared with PET/CT, enhancing clinical diagnosis ( Fig. 4 ) and facilitating better patient management in 80% of the total studied cases. Notably, in Fig. 5C–E , PET/MRI provided crucial supplementary information regarding the relationship between a large hepatic mass and adjacent structures, such as the gallbladder, duodenum, CHD, and CBD, which was only partially visualized on PET/CT images ( Fig. 5A, B ). Furthermore, PET/MRI enhanced the identification of supplementary subcentimeter liver lesions as can be seen in Figs. 2 and 3 , underscoring its superior sensitivity in lesion detection compared with PET/CT.

In the patient cohort, PET/MRI could detect an impressive 40 additional lesions as opposed to PET/CT. These lesions were as small as 2 mm in long-axis diameter. Additionally, PET/MRI displayed a higher TLR of the SUV as compared with PET/CT, which had a significant impact on the final reports and influenced therapeutic decisions. The findings from PET/MRI notably affected the response assessment category in 5 cases and defined malignant hepatic lesions on staging/restaging scans in 10 out of 15 cases. The study concludes that PET/MRI offers enhanced diagnostic capabilities over PET/CT, providing valuable insights into hepatic lesions and improving patient outcomes.

### MR Sequences Enhance Lesion Detectability in PET/MRI

Among the sequences included in the protocol, the T2 FSE FAT SAT sequence demonstrated superior delineation of small liver lesions. This sequence employs a T2-weighted fast spin-echo sequence with fat saturation, which effectively suppresses fat signal, thereby enhancing the visualization and characterization of lesions within the liver. Furthermore, the late arterial phase contrast imaging showed better delineation of small subcentimeter liver lesions as compared with early arterial phase contrast imaging.

The study emphasized the significant value of the spatial resolution of MRI and the complementary data acquired simultaneously by PET/MRI in evaluating lesion viability. The higher spatial resolution of MRI allows for clearer visualization of lesion characteristics and provides additional information for precise diagnosis. Additionally, advanced MR techniques such as DWI and ADC mapping contributed to clinical lesion diagnosis, further enhancing the diagnostic accuracy of PET/MRI.

## Discussion

Accurate identification of hepatic lesions is crucial for optimal therapy and improved patient outcomes. Hence, it becomes crucial to choose an imaging modality that offers the highest level of accuracy and precision. 10 Both PET-MR and PET-CT have become valuable tools in hepatic lesion detection, providing improved diagnostic capabilities. This extensive study thoroughly evaluated the efficacy of PET/MR and PET/CT in detecting and characterizing hepatic lesions, with a particular emphasis on 18F-FDG–avid lesions. Although many studies have suggested PET/CT to be more sensitive than conventional CT in detecting hepatic lesions, 13 due to several limitations, PET/MR scans are increasingly recommended to evaluate the efficiency and precision of hepatic lesion detection. These scans are suggested to be done alone or as a delayed scan after the initial PET/CT examination.

PET/MR stands out as a powerful and advanced tool for lesion detection, particularly in the realm of initial tumor staging. This hybrid imaging modality exhibits a synergistic effect, combining the metabolic information from PET with the detailed anatomical and soft tissue characterization provided by MRI. 14 One major benefit of PET/MR compared with PET/CT is the substantial decrease in radiation exposure, making it a great choice for patients who require multiple imaging sessions. The combined PET/MR images demonstrate a notable enhancement in sensitivity and specificity for identifying malignancies in comparison to conducting separate MRI and PET scans. Accurate tumor staging and treatment planning rely heavily on this enhanced extent of precision. In addition to tumor staging, PET/MRI shows promising potential in assessing local lesions, leveraging the innate benefits of MRI. The extensive anatomical information provided by MRI improves the accuracy of evaluating the extent of invasion, giving clinicians a thorough understanding of the scope of the lesion. 10

In the context of hepatic neoplastic lesions, a common application of PET/MRI, complete and accurate detection becomes paramount, especially in diseases such as colon or rectal cancer, where therapeutic decisions hinge on factors such as the size and number of lesions. The amalgamation of a diagnostic multiphase MRI of the liver, which is widely accepted as the current standard of care, with PET evaluation offers a distinct and valuable opportunity. This combination not only provides enhanced sensitivity and specificity but also enables a more thorough evaluation of the likelihood and viability of distant disease in the liver. 9

This study demonstrated that PET/MRI, when compared with PET/CT, exhibited superior performance in terms of lesion delineation and anatomic allocation of PET-positive findings. It is worth mentioning that the addition of the diagnostic T2 FSE FAT SAT sequence in PET/MR was critical in achieving better delineation of small liver lesions. PET/MRI showcased enhanced capabilities in precisely defining the boundaries and anatomical locations of the lesions over PET/CT. This advantage in anatomic delineation is consistently reflected in the improved classification of hepatic lesions, emphasizing the clinical significance of PET/MRI in providing more accurate spatial information for these lesions.

It is worth mentioning that PET/MRI yielded valuable additional insights in 87% of cases (13 out of 15), enhancing our general comprehension of the disease. In 73% of patients (11 out of 15), PET/MRI managed to successfully detect additional lesions that were not identified by PET/CT. This highlights the enhanced sensitivity of PET/MRI in detecting lesions, which is essential for precise staging and planning of treatment. The additional lesion findings changed treatment planning for 20% of the patients where PET/CT only detected one lesion, whereas PET/MRI detected multiple lesions leading to the change in treatment and patient management. In some patients, detection of additional lesions on PET/MRI altered treatment plans, including surgical management.

The enhanced lesion delineation observed in 80% of the total studied cases further emphasizes the superiority of PET/MRI over PET/CT. This improved delineation not only helps with the precise identification of abnormalities but also has important implications for clinical diagnosis and subsequent patient care. Particularly noteworthy is the identification of supplementary subcentimeter liver lesions facilitated by PET/MRI, emphasizing its capability to detect smaller lesions that might be overlooked by other imaging modalities.

When it comes to lesion detection, PET/MRI proved to be more effective than PET/CT by detecting an additional 40 lesions in the patient group. A tiny lesion, measuring only 2 mm in long-axis diameter, has been effectively detected, illustrating the remarkable sensitivity of PET/MRI in identifying even the smallest abnormalities. Across 15 patients, the average long-axis diameter of small lesions measured 3.4 mm, with a standard deviation of 1.3 mm. This emphasizes the accuracy of PET/MRI in analyzing lesions of different sizes. In addition, the data we acquired revealed that PET/MRI had a higher TLR compared with PET/CT. This metric is critical in assessing the metabolic activity of lesions, providing valuable insights into their biological characteristics.

The reconstruction of PET datasets using a 3D OSEM and HYPER Deep Progressive Reconstruction Algorithm, along with the automatic generation of a four-compartment-model attenuation map (μ-map) based on a water-fat-imaging sequence with breath gating for PET/MRI, contributed to the accuracy and reliability of the results obtained. This robust methodology ensures precise attenuation correction, enhancing the overall diagnostic capability of PET/MRI in hepatic lesion evaluation. In conclusion, the comprehensive results of this study affirm that PET/MRI is a superior imaging modality, offering enhanced lesion detection, delineation, and characterization compared with PET/CT in the evaluation of hepatic lesions.

A few limitations need to be acknowledged in this study. First, the use of FDG as a tracer is not tumor-specific, necessitating an understanding of physiological variations in FDG uptake and potential pitfalls and artifacts. Nonspecific FDG uptake can lead to false positives or negatives, affecting the accuracy of lesion characterization. Second, the study primarily consists of a single-center case series, limiting the generalizability of findings to broader populations. 15 Multicenter studies could provide more robust evidence of the general applicability of PET/MRI in hepatic lesion evaluation. Finally, in the detection of 18F-FDG–negative lesions, the performance of the CT versus MRI components may differ significantly, potentially influencing the overall sensitivity and specificity of the imaging modality. Awareness of these limitations is crucial for the judicious interpretation of results and underscores the need for further research to address these challenges.

## Conclusion

In conclusion, our study provides compelling evidence supporting the significant advantages of PET/MRI over PET/CT in the detection and characterization of hepatic lesions. One notable advantage is the substantial reduction in ionizing radiation exposure by 80%, reinforcing PET/MRI as a safer alternative for patients requiring repeated imaging sessions. Additionally, the study revealed higher detection rates and more precise characterization of small malignant liver lesions with the PET/MRI combination, emphasizing its superior clinical utility.

The incorporation of the T2 FSE FAT SAT MR sequence in PET/MRI demonstrated a higher rate of concordant findings compared with PET/CT. This heightened concordance, particularly in the context of smaller lesions, not only enhances diagnostic certainty but also has direct implications for patient management. PET/MRI emerges as a powerful imaging tool, offering a more comprehensive and accurate assessment that directly impacts patient diagnosis, staging, and subsequent therapeutic strategies. 9

These findings underscore the potential of PET/MRI to serve as a transformative imaging technology in oncologic imaging, providing clinicians with enhanced diagnostic confidence and valuable insights for individualized patient care. As we move forward, the utilization of PET/MRI, with its notable reduction in radiation exposure and improved diagnostic performance, has the potential to reshape the landscape of hepatic lesion evaluation, influencing clinical decision-making and ultimately improving patient outcomes. Further research and broader multicenter studies will be crucial to solidify these findings and establish PET/MRI as a standard in hepatic lesion detection and characterization.
